# Synergistic effects of core@double-shell structured magnesium hydroxide microcapsules on flame retardancy and smoke suppression in flexible poly(vinyl chloride)†

**DOI:** 10.1039/d1ra09030e

**Published:** 2022-01-20

**Authors:** Jingshui Xu, Haiying Yang, Zibo Luo, Dang Wu, Gengyu Cao

**Affiliations:** Shantou Guangyou-Malion New Materials Research Institute, Guangdong University of Petrochemical Technology Maoming 525000 China gyucao@gdupt.edu.cn

## Abstract

In order to develop an effective flame retardant for poly(vinyl chloride) (PVC), a core@double-shell structured magnesium hydroxide@9,10-dihydro-9-oxa-10-phosphaphenanthrene-10-oxide@melamine formaldehyde resin (MH@DOPO@ MF) encapsulated flame retardant was prepared. Its flame retardancy and smoke suppression effects in flexible PVC were investigated. Results show that the PVC/10 wt% MH@DOPO@MF composite has the best flame retardancy and smoke suppression performance in comparison with pure flexible PVC and the PVC/20 wt% MH composite. The limiting oxygen index (LOI) of the PVC/10 wt% MH@DOPO@MF composite was ∼30.8%, achieving a V-1 rating in the UL-94 test. MH@DOPO@MF in PVC remarkably increases the yields of the residual char and drastically decreased the heat release rate (HRR), total heat release (THR), smoke production rate (SPR) and total smoke production (TSP). The mechanical property testing showed that MH@DOPO@MF had slight damage on the tensile strength and elongation at break of PVC. This is ascribed to the synergistic flame-retardant effects of MH coordination with DOPO and MF. The present work demonstrates that the core@double-shell structured microcapsule (MH@DOPO@MF) prepared in this efficient manner has good flame retardancy and smoke suppression, and may provide a candidate flame retardant for applying in flexible PVC.

## Abstract 2

It can be concluded that MH@DOPO@MF incorporated in flexible PVC improves its flame retardancy. The possible synergistic flame-retardant mechanism of PVC/10 wt% MH@DOPO@MF composite was proposed.

## Graphical abstract

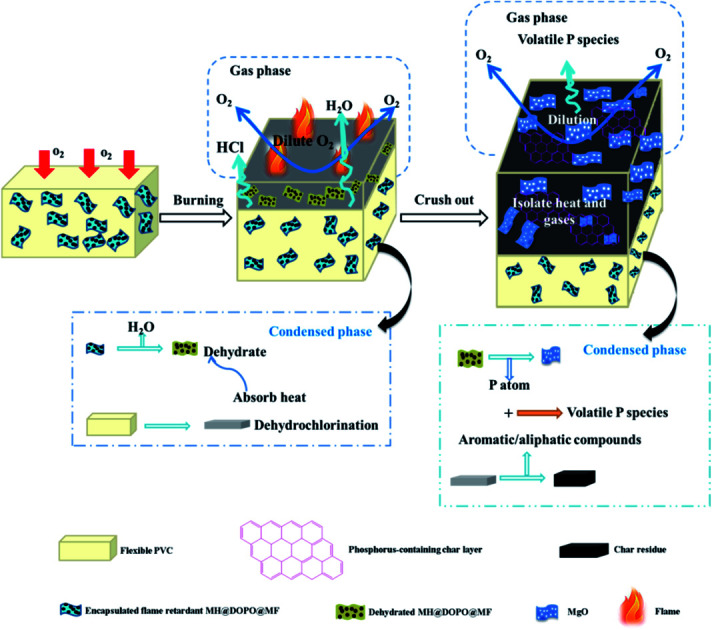

## 1. Introduction

Flexible poly(vinyl chloride) (PVC) is widely used in the fields of electrical insulation, sealing, toys and medical devices, and is an ideal substitute for rubber and elastomer materials with low cost and easy fabrication.^1,2^ However, most flexible PVC composites are flammable, generating toxic gases and heavy black smoke during combustion, which dramatically limit their practical applications, especially in the electrical fields.^3,4^ Therefore, the improvement of flame retardancy and smoke suppression will make flexible PVC materials more suitable.

As the global health and regulatory scrutiny is concerned, a wide variety of environment-friendly, cost-efficient and halogen-free flame retardants and smoke suppressants have been proposed and applied in PVC composites.^5–8^ Among magnesium hydroxide (MH), aluminum trihydrate (ATH), zinc hydroxystannate (ZHS), zinc stannate (ZS), *etc.*, as a typical representative, MH has been extensively used as an environment-friendly halogen-free flame retardant in PVC materials because of its high decomposition temperature.^9–13^ Numerous works dealing with the flame retardance mechanism of MH have been reported,^14–16^ and the results have confirmed that the flame retardancy and smoke suppression properties of MH are mainly attributed to the following two factors: firstly, the decomposition of MH produces water vapor that dilutes the combustible gas in the gas-phase at above 300 °C, secondly, the physical barrier composed of magnesium oxide (MgO) layers is formed in the condensed-phase during the decomposition process of MH. However, it is noted that MH shows low flame-retardant efficiency. Thus, in order to meet the requirements of flame retardancy and smoke suppression of polymeric composites, the addition of MH exceeds 50 wt%, which greatly damages the processability and mechanical properties of composites.^17–19^ Consequently, how to better construct a compounded system to modify MH with environment-friendly and halogen-free flammability so as to solve its low flame retardant efficiency and poor dispersity in polymer matrix, which is of great significance for its practical applications in polymeric composites.

Currently, for solving the deficiency of single component flame retardants, a great interest is focusing on the synergistically flame retarding effects of novel flame retardants with inorganic metal (aluminum, magnesium and calcium, *etc.*) hydroxide.^20–22^ So it is desirable to use synergistic flame retardant technology to obtain novel flame retardance PVC systems with low-loading and excellent flame retardance efficiency. For example, Dang L., *et al.*^23^ successfully prepared the magnesium hydrate (MH)@molybdenum trioxide (MO) flame retardant and incorporated it into flexible PVC. Resultants showed that the as-prepared MH@MO had better synergistic flame retardance efficiency for flexible PVC in comparison with that of any single component of them. Recently, Meng W. H., *et al.*^24^ prepared an effective bio-based flame retardant compound consisting of MH doped with tin phytate and zinc tannate, and results showed that the obtained compounded flame retardant incorporation into flexible PVC exhibited an excellent flame retardancy and smoke suppression properties. This is due to the fact that tin phytate and zinc tannate synergistically catalyze the formation of continuous and high-density char residues in the combustion process of PVC. However, the main defects of synergistic flame retardant compounds are that they tend to serious agglomerate and poor dispersibility in polymers. In recent years, it is found that microencapsulation is an effective method to overcome the above-mentioned deficiency.^25^ Microencapsulation as a kind of protection technology which widely used in pesticides, paint, textiles, *etc.*, can encapsulate some organic or inorganic substances to form different size microcapsules, so as to improve the interfacial interaction and compatibility with polymer matrices.^11,24^ Hence, the microencapsulation of synergistic flame retardant compounds could make them better dispersed in polymer matrices.^26,27^

Among the halogen-free polyphosphate flame retardants, 9,10-dihydro-9-oxygen-10-phosphoheterophene-10-oxide (DOPO) has been found to have excellent gas-phase high efficiency flame retardance.^28^ Compared with other linear low molecular weight aliphatic phosphates and phosphorous compounds, DOPO presents higher thermal stability, chemical stability, excellent flame retardancy and low toxicity owing to the existence of diphenyl cyclophosphate groups in its molecular structure. Furthermore, it has high char forming ability and flame-retardant effect in condensed-phase.^29^ Besides, owing to the active P–H bonds in the molecule of DOPO, it can react better with unsaturated groups, *i.e.*, epoxy group, Schiff base, carbon–nitrogen double bond (C=N) and triple (C≡N), carbon–carbon double bond (C=C), *etc.*, so that various types of DOPO-based synergistic flame retardants and smoke suppressants can be synthesized with others.^30,31^ In addition, melamine formaldehyde (MF) resin is also an environment-friendly N-based flame retardant and its thermal stability is above 380 °C.^32^ Although MF resin dose not react directly with MH and DOPO, its prepolymer can be adsorbed to MH and DOPO surface by a sol–gel method so as to form shell on the surface of MH and DOPO. It would form a magnesium–phosphorus–nitrogen (Mg–P–N) synergetic system with MH and DOPO to show better flame retardancy and smoke suppression properties than those of single core–shell encapsulated flame retardants.^1,11^ Theoretically, the combination of MF, DOPO and MH displays a synergetic effect in improving the flame retardancy and smoke suppression properties of polymeric composites. Because the decomposition of Mg–P–N system can form compact protective layers by an endothermic process to inhibit oxygen (O_2_) as well as heat to go into material bulk.^20,25,32^ Nevertheless, there are few reports on the environment-friendly and cost-efficient halogen-free synergistic flame retardant of the combination for MH, DOPO and MF, and its flame retardancy and smoke suppression mechanism applied in PVC materials.

Herein, in this paper, a type of core@double-shell structured (MH@DOPO@MF) microcapsule was developed in order to reduce the dosage and cost of flame retardants. And then the as-prepared MH@DOPO@MF containing various flame-retardant elements such as magnesium (Mg), phosphorus (P) and nitrogen (N) is synchronously introduced into flexible PVC materials to look forward to obtain very prominent synergistic flame retardancy and smoke suppression effects. Moreover, the mechanical properties, flame retardancy and smoke suppression mechanism of PVC/MH@DOPO@MF composites were characterized by multiple test instruments such as electronic universal testing instrument, UL-94 horizontal burning level (UL-94), limiting oxygen index (LOI), cone calorimeter test (CCT), scanning electron microscope (SEM), pyrolysis-gas chromatography/mass spectrometry (Py-GC/MS), and thermogravimetry analysis coupled with Fourier transform infrared spectroscopy (TGA-FTIR) tests.

## 2. Experimental

### 2.1. Materials and chemicals

Magnesium hydroxide [Mg(OH)_2_, nanoscale hexagonal laminate structure] was provided by Jilin Taxus Technology Development Co., Ltd (Jilin, China). γ-(2,3-propylene epoxide)propyl trimethoxysilane (γ-MPS) was purchased by Shandong Leon New Material Technology Co., Ltd (Shandong, China). 9,10-Dihydro-9-oxa-10-phosphaphenanthrene-10-oxide (DOPO, reagent grade) was provided by Shouguang Weidong Chemical Co., Ltd (Shouguang, China). Acetic acid [analytic reagent (AR)], melamine, formaldehyde (AR, 37–40%) sodium carbonate (Na_2_CO_3_, AR) were purchased by Shenzhen Zhongyue Chemical Co., Ltd (Shenzhen, China). Sodium dodecyl sulfate (SDS, AR) and polyoxyethylene octylphenol ether (OP-10, AR) was obtained from Shanghai Chlor Alkali Chemical Co., Ltd (Shanghai, China). The materials used PVC (TMP-31, *K* = 70, average polymerization degree for 1230–1430) were purchased by Xinjiang Tianye Company (Xinjiang, China). Ba and Zn-containing thermal stabilizer (CZ-707) and dioctyl phthalate (DOP, plasticizer) were purchased from Jinan Hede Chemical Co., Ltd (Shangdong, China). All other reagents (AR) were of analytical grade and were provided from Xilong Chemical Co., Ltd (China). Deionized water was used for all experiments.

### 2.2. Preparation of MH@DOPO

**Scheme 1:**
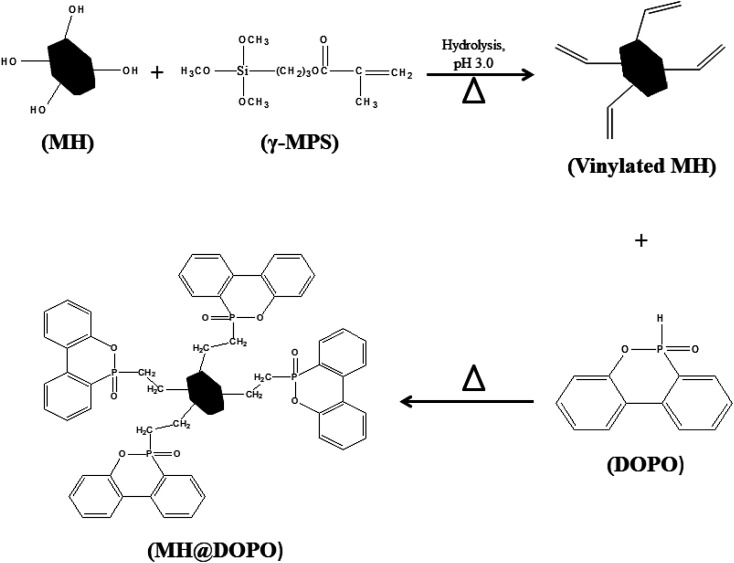
Schematic illustration for the preparation of MH@DOPO.

As shown in Scheme 1, the preparation of MH@DOPO was conducted on the basis of a previous procedure reported by Liu S. P.^33^ with a slight modification. Firstly, 30 g of MH was dispersed in 100 mL ethanol solution in a 1000 mL three-necked reactor equipped with a stirrer and a thermometer. Secondly, a calculated amount of γ-MPS solution (*m*_ethanol_ : *m*_H_2_O_ : *m*_γ-MPS_ = 100 : 15 : 1) was added into a 1000 mL flask, and its pH value was slowly tuned to ∼3.0 by adding acetic acid at 25 °C for stirring 30 min. Subsequently, it was added dropwise to the MH dispersion, and then heated up to 60 °C for 2 h with continuous stirring under N_2_. Thirdly, 20 g of DOPO was dissolved in a moderate amount of anhydrous ethanol in a 100 mL flask, and then was slowly dropped into the reactor and heated up to 90 °C for 4 h. After the reaction, the sample was centrifuged and successively washed with anhydrous ethanol and deionized water for 5 times so as to remove the excess reagent. Finally, the obtained MH@DOPO was freeze-dried for 24 hours and carefully stored for future use.

### 2.3. Preparation of MH@DOPO@MF microcapsule

Preparation of MH@DOPO@MF microcapsule was conducted on the basis of a previous procedure reported by Zhang B., *et al.*^11^ Briefly, 12.6 g of melamine, 20.29 g of formaldehyde and 20.76 of butyl alcohol were mixed in 20 mL deionized water in a 500 mL three-necked reactor equipped with a stirrer and a thermometer. The pH value of the mixture was slowly tuned to ∼8.5 using Na_2_CO_3_ aqueous solution (10 wt%), and then stirred at 70 °C until it became clearly transparent so that MF pre-polymer solution was obtained. Then the above MF prepolymer, 0.75 g of SDS, 0.25 g of OP-10 and 42.92 g of the as-prepared MH@DOPO were dispersed into 100 mL deionized water in a 500 mL three-necked reactor equipped with a stirrer, a thermometer and a condensation reflux device. The reactor was heated up to 60 °C under vigorously stirring until forming an emulsion. Obtained emulsion was cooled gradually to 25 °C, and then its pH value was slowly tuned to ∼9.0 by Na_2_CO_3_ aqueous solution (30 wt%) and kept for 30 min. Eventually, the obtained sample was filtered through a Buchner funnel and rinsed with deionized water 5 times, and then dried at 70 °C overnight.

### 2.4. Preparation of flexible PVC composites

According to the design formula, 100 g of PVC resin, 50 g of plasticizer DOP, 2.5 g of stabilizer containing Ba and Zn (Z-707) and calculated amount of MH@DOPO@MH were mixed mechanically at 25 °C for 30 min. After mixing, samples were vacuumed evenly for 5 min, repeat 3 times to completely remove bubbles. The slurry was uniformly coated on polytetrafluoroethylene (PTFE) film at 105 °C for 7 min. After drying, the PVC/10 wt% MH@DOPO@MF sample was hot-pressed at 165 °C for 1 min and 7.5 MPa for 3 min. After cooling, obtained samples were cut to desired size. For comparison, pure flexible PVC and its PVC/20 wt% MH composites were prepared by the same method.

### 2.5. Characterization and analysis

The limiting oxygen index (LOI) value of the sample was determined by JF-3 oxygen index test instrument (Jiangning, China) in accordance with GB/T 2406.2-2009 standard. The size was of 100 × 6.5 × 3 mm^3^.

The UL-94 vertical burning test (UL-94) of the sample was determined by CFZ-3 instrument (Jiangning, China) according to ANSI/UL 94-2013. The size was of 130 × 13 × 3.2 mm^3^. Each group of samples was tested 5 times in parallel to ensure the reliability and repeatability of the data. UL-94 test results were classified according to the combustion grades of V-0, V-1 and V-2.

Cone calorimetric test (CCT) of the sample was determined by a Fire Testing Technology Limited (FTT) cone calorimeter (JCZ-2, Jiangning, China) according to ISO 5660-2015. The size was of 100 × 100 × 3 mm^3^. It was irradiated at an external heat flux of 35 kW m^−2^. The experimental error of cone calorimeter data is ∼5%. The char residues of samples after CCT were first photographed with a digital camera and subsequently analyzed by FTIR, X-ray diffraction (XRD) and SEM, respectively. XRD measurements of the char residues were performed on Bruker D8 Advance X-ray diffractometer (STADI P, Germany) at room temperature. The diffraction patters were determined over a range of diffraction angles of 1.5° to 70° at a scanning rate of 3° min^−1^ with a sampling width of 0.02. FTIR spectra of the char residues and samples were recorded with KBr powder on an FTIR spectrometer. FTIR spectra were obtained in the region from 4000 cm^−1^ to 400 cm^−1^. Morphological structures of the char residues were taken on a JEOL S-4800 (Hitachi, Japan) at 10 keV or 15 keV. The specimens were coated with a conductive layer of gold prior to testing.

Py-GC/MS test was carried out on a gas chromatography (Agilent 6980 N) and mass spectrometer (Agilent 5975). About 0.3 g of sample was heated from 25 °C to 750 °C with a rate of 20 °C min^−1^ in helium. The temperature program of the capillary column (HP-5MS) of GC was as following: 50 °C held for 5 min, and then temperature increased to 260 °C with a rate of 10 °C min^−1^, and 260 °C held for 10 min. The detection of mass spectra was performed using a NIST library.

TGA-FTIR test was performed using a Netzsch TG 209-F3 (Germany) thermogravimetry coupled with a Nicolet 6700 FTIR spectrometer. About 10 mg of sample was heated from 25 °C to 700 °C with a hating rate of 20 °C min^−1^ under N_2_. FTIR spectra were obtained in the region from 4000 cm^−1^ to 500 cm^−1^.

Tensile strength of sample was measured according the Chinese standard method (GB T1040-92) with WDW-10D electronic universal testing instrument (Jiannan Test Metal Group Limited Company) at a strain speed of 20 mm min^−1^. All the samples were performed at room temperature. Each tensile test was repeated at least five times and the results were averaged.

## 3. Results and discussion

### 3.1. LOI and UL-94 analysis

LOI and UL-94 tests monitor the flammability of samples. Standard test samples were prepared by a melt-blending encapsulated MH@DOPO@MF with the flexible PVC to intuitively demonstrate the flammability of samples. As comparison, similarly test samples of pure flexible PVC and its PVC/MH were also fabricated by the same procedure. And the corresponding data are summarized in Table 1. The pure flexible PVC is combustible and its LOI value is only 21.9% with no rating in the UL-94 test. The LOI value of PVC/20 wt% MH is 23.8%, which slightly higher than that of pure flexible PVC. Significantly, the LOI value of PVC/10 wt% MH@DOPO@MF is increased to 30.8%, which is 9.1% higher than that of pure flexible PVC. Hence, it can be seen that DOPO@MH@MF plays an important role in endowing PVC with good flame retardance.

**Table 1:** LOI and UL-94 test data of pure flexible PVC, PVC/20 wt% MH and PVC/10 wt% MH@DOPO@MF

Sample	LOI/%	UL-94^a^	Dripping
Flexible PVC	21.8 ± 0.1	NR	Very intense
PVC/20 wt% MH	24.7 ± 0.1	V-2	Dropping
PVC/10 wt% MH@DOPO@MF	30.9 ± 0.1	V-1	No dripping

a NR indicates no level.

The detailed data of UL-94 are listed in Table 1. During the tests, pure flexible PVC burns fiercely after ignition. In comparison with the PVC/20 wt% MH, it burns for ∼7 s after the first ignition, and there is serious dripping phenomenon in the second ignition. It's worth noting that, even cotton fibre can be ignited by these drops, meaning that PVC/20 wt% MH with V-2 rating in the UL-94 test. Particularly, PVC/10 wt% MH@DOPO@MF burns slowly for ∼5 s after the first ignition and extinguishes ∼7 s after the second ignition. More importantly, no drips are observed throughout this test, meaning that PVC/10 wt% MH@DOPO@MF has reached the V-1 rating. Obviously, MH@DOPO@MF introduction into PVC materials performs better flame retardance in the UL-94 tests, indicating that it is difficult to burn in comparison with pure flexible PVC. Therefore, MH@DOPO@MF in PVC composite system plays excellent synergistic flame retardance.

### 3.2. Morphology at different temperatures

**Fig. 1:**
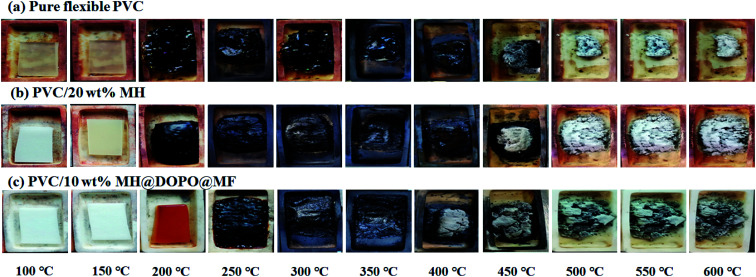
Digital photographs of samples after storage at different temperatures for ∼15 min in a muffle furnace, (a) pure flexible PVC, (b) PVC/20 wt% MH, (c) PVC/10 wt% MH@DOPO@MF.

The morphologies of pure flexible PVC, PVC/20 wt% MH and PVC/10 wt% MH@DOPO@MF composites were observed in different temperatures for 15 min in a muffle furnace, and photographed with a digital camera. Obtained results are shown in Fig. 1. It can be seen that, pure flexible PVC has changed color at 100 °C and the char residue in the crucible is 8.7 wt% at 600 °C. The PVC/20 wt% MH has changed color in 150 °C and the char residue rate in the crucible is 20.9 wt% at 600 °C, containing white MgO powders. The results showed that MH had low flame retardance when it was added into polymer matrices below 50 wt%.^23^ The PVC/10 wt% MH@DOPO@MF has formed continuous carbon layer at 350 °C and its char residue rate in the crucible is 26.3 wt% at 600 °C. In summary, the as-prepared MH@DOPO@MF has exerted a synergistic flame-retardant efficiency.

### 3.3. Flame retardancy and smoke suppression properties assessed by CCT

#### 3.3.1. Evaluation of fire hazard

**Fig. 2:**
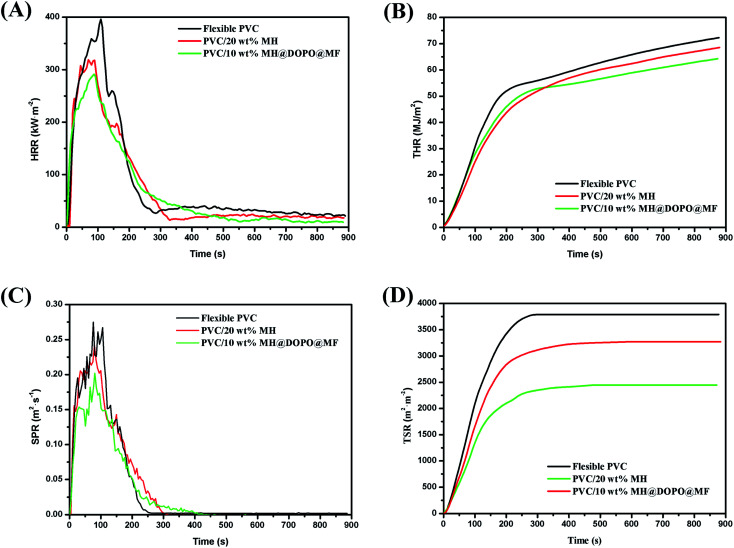
Cone calorimetric test curves of pure flexible PVC, PVC/20 wt% MH and PVC/10 wt% MH@DOPO@MF, (A) heat release rate (HRR), (B) total heat release (THR), (C) smoke production rate (SPR) and (D) total smoke production (TSP).

CCT on account of the oxygen consumption principle, is also an important method evaluate the fire behavior of polymers.^24,34^Fig. 2 and Table 2 present peak heat release rate (PHRR), heat release rate (HRR), total heat release (THR), peak smoke production rate (PSPR), smoke production rate (SPR), total smoke production (TSP) and the time to ignition (TTI).

**Table 2:** CCT data for pure flexible PVC, PVC/20 wt% MH and PVC/10 wt% MH@DOPO@MF at a heat flux of 35 kW m^−2^

Sample	TTI (s)	PHRR (kW m^−2^)	THR (MJ m^−2^)	PSPR (m^2^ s^−1^)	TSP (m^2^ m^−2^)
Flexible PVC	14 ± 1	396 ± 9	72.6 ± 0.6	0.275 ± 0.007	3680 ± 32
PVC/20 wt% MH	18 ± 1	318 ± 8	68.5 ± 0.5	0.241 ± 0.010	3271 ± 40
PVC/10 wt% MH@DOPO@MF	22 ± 1	284 ± 6	64.2 ± 0.5	0.198 ± 0.005	2438 ± 25

**Fig. 3:**
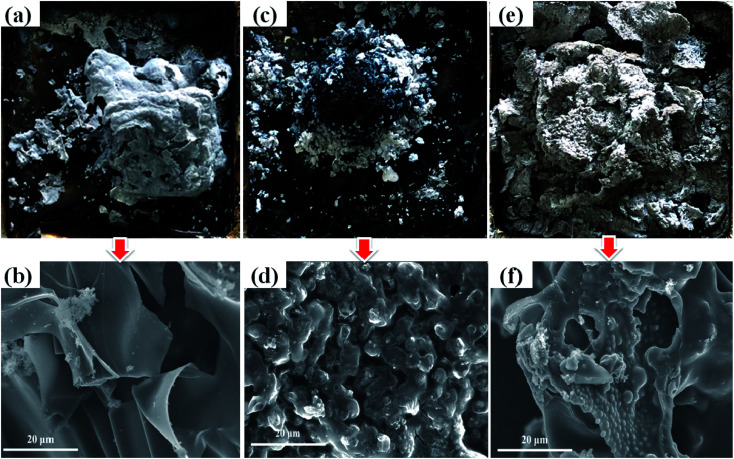
Digital photographs and SEM images of residual char of samples, (a and b) pure flexible PVC, (c and d) PVC/20 wt% MH, (e and f) PVC/10 wt% MH@DOPO@MF.

Heat release rate is the most important performance parameter to characterize fire intensity, that refers to the heat release rate per unit area under the preset incident heat flux after the material is ignited.^35^ The larger the number, the greater heat is released from the combustion of materials, the resulting fire hazard is greater. As shown in Fig. 2(A), all experimental samples have burned intensely within 250 s of ignition. Pure flexible PVC has a sharp burning after being ignited and its HRR curve presents a sharp peak. The PHRR value of pure flexible PVC is 396 kW m^−2^ at 108 s. After that, its HRR value gradually decreases. Besides, the PHRR values of PVC/10 wt% MH@DOPO@MF and PVC/20 wt% MH are 284 kW m^−2^, 318 kW m^−2^, respectively. After reaching the maximum value, the HRR value of PVC/10 wt% MH@DOPO@MF decreases rapidly. This phenomenon is due to the endothermic reaction of MH dehydration, which is beneficial to reduce HRR.^24^ In addition, the phosphoric acid and its analogues produced during the thermal decomposition of the phosphate esters in MH@DOPO@MF facilitated the formation of a more stable char residue for preventing the transmission of heat.^1,30^ The TTI value of PVC/10 wt% MH@DOPO@MF is corresponding to a 57.1% prolonged compared to that of pure flexible PVC, which is very important for the fire rescue. This was consistent with the LOI test results (seen in Table 1). Because the formation of carbonization layer is formed from the rapid cross-linking of the synergistic compounded system, resulting in the heat release rate rapidly decreased. For further evaluating their carbonization effects, the morphology of the char residues is analyzed (seen in Fig. 3). As seen from Fig. 2(B), the THR of PVC/10 wt% MH@DOPO@MF shows a further decrease compared to that of PVC/20 wt% MH, and it is ∼64.2 MJ m^−2^ corresponding to a 12% reduction to pure flexible PVC (∼72.6 MJ m^−2^). The reason for THR decrease is probably caused by the increase of residual char at high-temperature.^36^

The smoke production in the process of fire is another key parameter.^37^ The investigated resultants indicate that asphyxiation is considered to be an important cause of death in actual fires.^38^ Especially, flexible PVC in the combustion process would produce a cast amount of dense and black smoke and toxic gases. And their negative effects are actually more serious than heat release. As shown in Fig. 2(C), the SPR curve of pure flexible PVC is similar to its HRR curve, and its PSPR value is ∼0.275 m^2^ s^−1^ at 108 s. In comparison, the PSPR value of PVC/10 wt% MH@DOPO@MF is clearly lower than that of pure flexible PVC and decreases from ∼0.275 m^2^ s^−1^ to 0.198 m^2^ s^−1^. However, the PSPR value of PVC/20 wt% MH is ∼0.241 m^2^ s^−1^, showing a slight decrease as compared with that of pure flexible PVC. Fig. 2(D) shows that the TSP curves of pure flexible PVC, PVC/20 wt% MH and PVC/10 wt% MH@DOPO@MF. Table 2 presents that ∼3271 m^2^ m^−2^ and 2438 m^2^ m^−2^ smoke is released from PVC/20 wt% MH and PVC/10 wt% MH@DOPO @MF, which is ∼11.1% and 33.8% lower than that of pure flexible PVC (∼3680 m^2^ m^−2^), respectively. So, MH@DOPO@MH incorporation into PVC materials performs excellent smoke suppression.

#### 3.3.2. Morphology analysis of residual char

To further investigate the flame retardance mechanism, the microstructures of the char layers and the morphologies of the char residues of samples after cone calorimetry tests were measured by digital photo and SEM analysis.^38,39^ Results are presented in Fig. 3. Fig. 3(a) and (b) shows the digital and SEM pictures of pure flexible PVC after the cone calorimetry test. It can be seen that the char residue of pure flexible PVC is rather inhomogeneous, relatively loose structure with holes and cracks on the surface. Obviously, the insufficient char formation can no effectively protect the combustion inside PVC matrix, so consequently heat and flammable volatiles can penetrate the char layer into the flame zone. As shown in Fig. 3(c) and (d), the char residue of PVC/20 wt% MH is not as nonuniformity as that of pure flexible PVC, and there is more continuous consisting of evenly dispersed MgO, holes and cracks. Fig. 3(e) and (f) shows that the char residue of PVC/10 wt% MH@DOPO@MF has higher char yield and rich char residue in comparison with that of pure flexible PVC. And its char layer is rather compact and has certain porosity and thermal stability, which can effectively prevent heat and combustible volatiles transfer in the combustion process. Therefore, these results indicated that MH coordination with DOPO and MF could not only inhibit the heating and pyrolysis of PVC matrix in a wider temperature range, but also release water vapor in a wider temperature range, thus showing a better flame retardancy and smoke suppression effect.

#### 3.3.3. XRD analysis of residual char

**Fig. 4:**
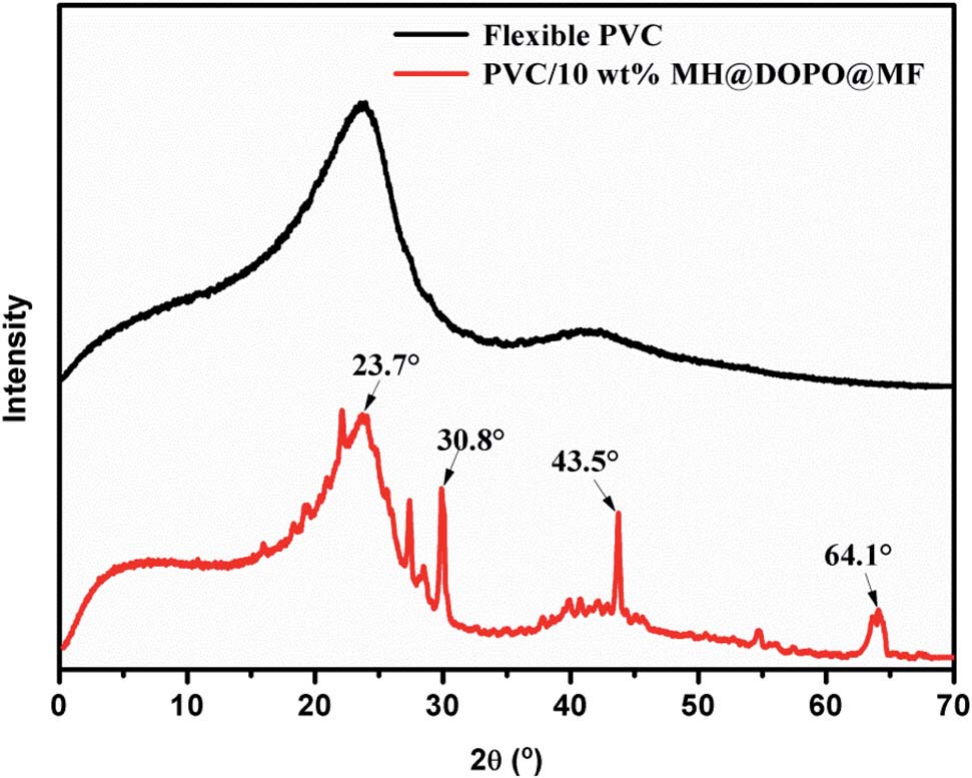
X-ray diffraction (XRD) patterns of residual char of pure flexible PVC and its PVC/10 wt% DOPO@MH@MF.

For further analyzing the composition of the residual char of samples after cone calorimetry tests, XRD was utilized to characterize the char in depth. And the relevant results of XRD are illustrated in Fig. 4. For pure flexible PVC, the crystalline peaks at 23.1° and 43.8° are caused by degradation productions of the barium stabilizer (CZ-707), which are also shown in the XRD pattern of PVC/10 wt% MH@DOPO@MF. The residual of PVC/10 wt% MH@DOPO@MF has several diffraction peaks at 2*θ* = 30.8°, 43.5° and 64.1°, which is MgO layer and carbon layer containing phosphorous. However, the above diffraction peak did not appear in the residue char of pure flexible PVC.

#### 3.3.4. FTIR analysis of residual char

**Fig. 5:**
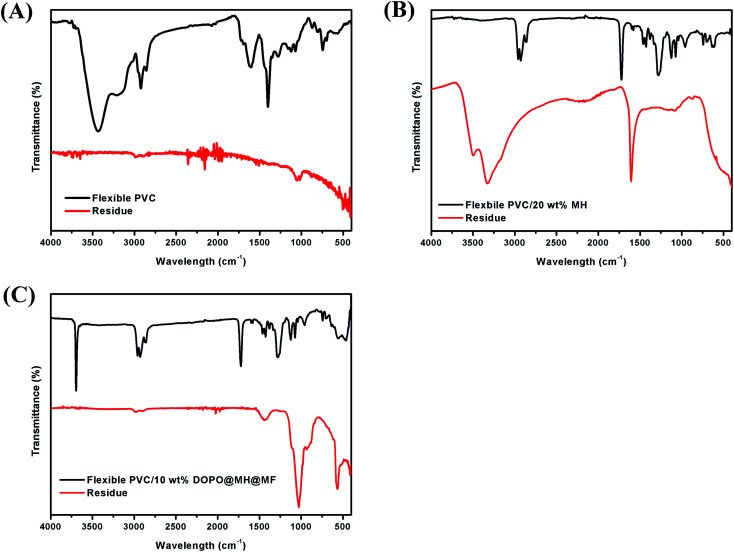
FTIR spectra of residual chars, (A) pure flexible PVC, (B) PVC/20 wt% MH, (C) PVC/10 wt% MH@DOPO@MF.

Fig. 5 showed the FTIR of pure flexible PVC, PVC/20 wt% MH and PVC/10 wt% MH@DOPO@MF composites, and their residual chars after cone calorimetric tests. As shown in Fig. 5(A), the decomposition of pure flexible PVC has been extremely thorough. For Fig. 5(B), the residual char of PVC/20 wt% MH has the infrared absorption peaks range from 400 cm^−1^ to 610 cm^−1^, corresponding to MgO characteristic absorption peaks. Basically, Fig. 5(C) shows that many infrared absorption peaks are found from residual char of PVC/10 wt% MH@DOPO@MF. Hence, these results indicated that partially effective structures had still been retained after PVC/10 wt% MH@DOPO@MF decomposed.

### 3.4. Py-GC/MS analysis

**Fig. 6:**
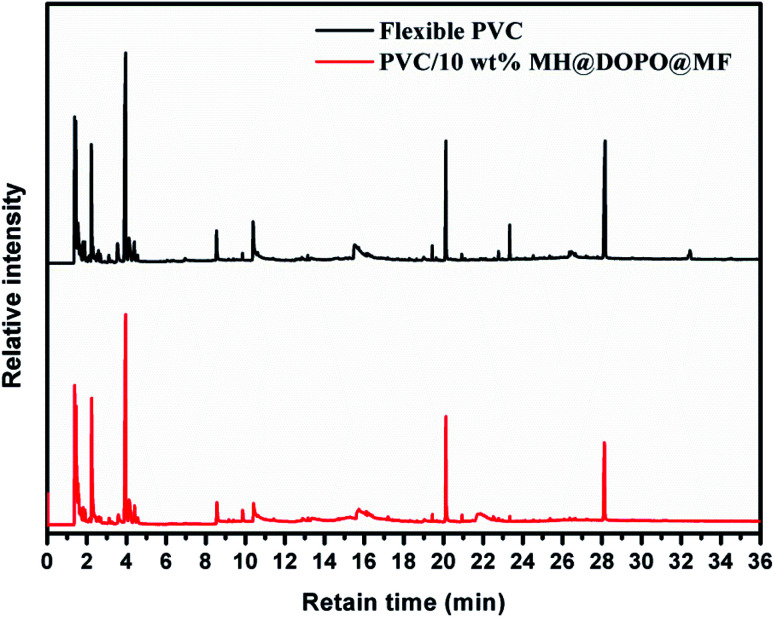
The Py-GC/MS of pure flexible PVC and its PVC/10 wt% MH@DOPO@MF.

For investigating the difference of pyrolysis behaviors between pure flexible PVC and its PVC/10 wt% MH@DOPO@MF composite, the Py-GC/MS tests were carried out, so as to make the flame retardance mechanism in the gas-phase of MH@DOPO@MF clarify. The pyrolysis temperature is set at 750 °C where MH@DOPO@MF has been broken completely. The corresponding results are presented in Fig. 6. And the structures corresponding to the peaks in the mass spectrum of pure flexible PVC and its PVC/10 wt% MH@DOPO@MF are listed in Tables 3 and 4. Generally, a chain reaction has happened in the gas-phase after polymer materials are ignited.^40,41^ During the combustion process, a large number of active free radicals H˙ and ˙OH are generated as PVC polymer chain broken, which can promote the chain breaking of PVC and accelerate its continuous decomposition. Thus, the quantity and strength of volatiles in pure flexible PVC gas are very high. However, some phosphorus fragments, P˙ and PO˙ radicals are generated as the P–C bonds in MH@DOPO@MF ruptured at high-temperature. Then they can terminate the chain reaction in the gas-phase by quenching ˙OH and H˙ radicals in the combustion region. Furthermore, the division of MH dilutes the hot air, cooling the surface of the pyrolysis zone and dehydrating it to form a carbonized layer. And the generated MgO could also adsorb free radicals. Hence, the quantity and intensity of vapor volatiles of PVC/10 wt% MH@DOPO@MF are significantly lower than those of pure flexible PVC.

**Table 3:** Compound identified in the Py-GC/MS of pure flexible PVC

No.	Retain time (min)	Molecular structure	Name	Molecular	*M*_w_ (g mol^−1^)
1	1.400	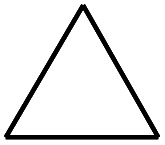	Furan, 2,5-dihydro-	C_3_H_6_	42
2	1.542	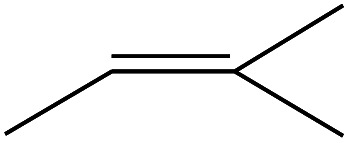	2-Butene, 2-methyl-	C_5_H_10_	70
3	1.633	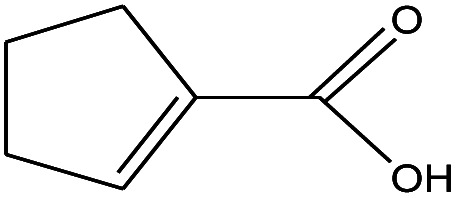	1-Cyclopentene-1-carboxylic acid	C_6_H_8_O_2_	112
4	1.892	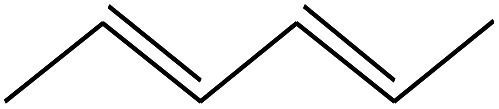	2,4-Hexadiene	C_6_H_10_	82
5	2.117	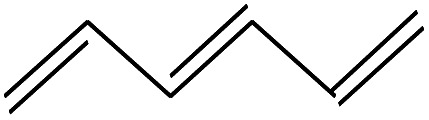	1,3,5-Hexatriene	C_6_H_8_	80
6	2.258	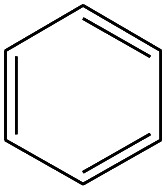	Benzene	C_6_H_6_	78
7	2.350	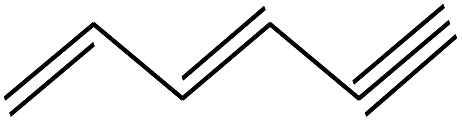	1,3-Hexadien-5-yne	C_6_H_6_	78
8	2.508	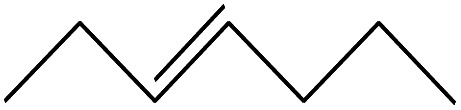	3-Heptene, (*E*)-	C_7_H_14_	98
9	2.583	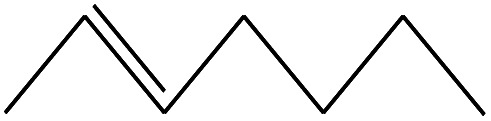	2-Heptene, (*E*)-	C_7_H_14_	98
10	3.108	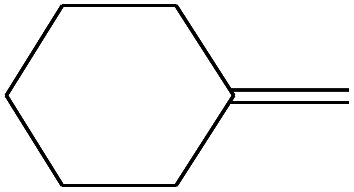	Cyclohexane, methylene-	C_7_H_12_	96
11	3.550	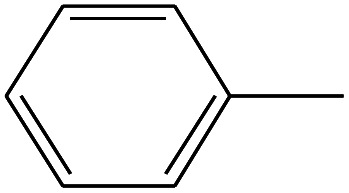	Toluene	C_7_H_8_	92
12	3.917	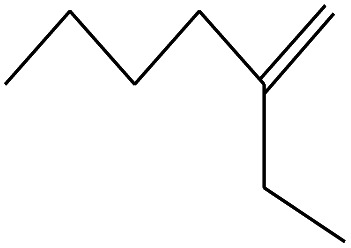	Heptane, 3-methylene-	C_8_H_16_	112
13	4.125	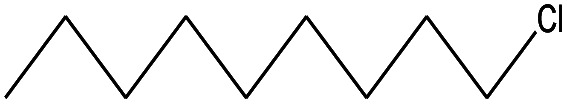	4-Octene, (*E*)-	C_8_H_17_Cl	112
14	4.383	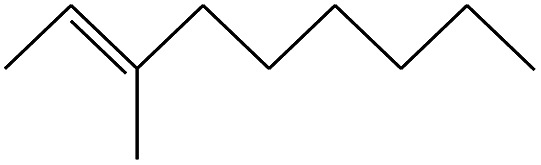	2-Heptene, 3-methyl-	C_8_H_16_	112
15	4.542	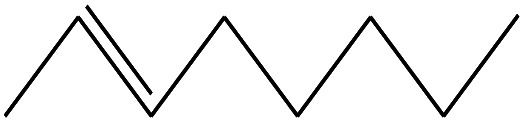	2-Octene	C_8_H_16_	112
16	6.967	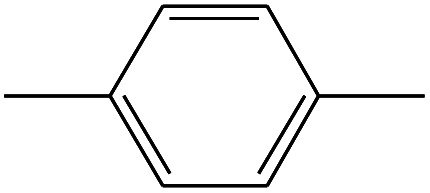	*p*-Xylene	C_8_H_10_	106
17	8.558	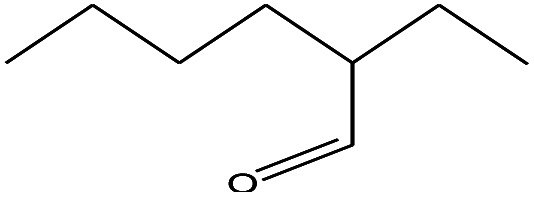	2-Ethylhexanal	C_8_H_16_O	128
18	9.833	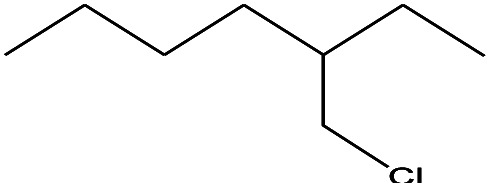	Heptane, 3-(chloromethyl)-	C_8_H_17_Cl	148
19	10.592	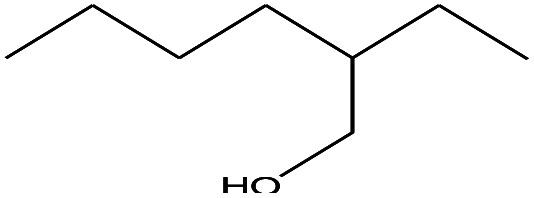	2-Ethyl-1-hexanol	C_8_H_18_O	130
20	12.875	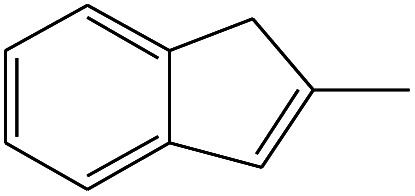	2-Methylindene	C_10_H_10_	130
21	13.342	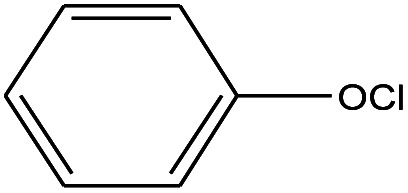	Phenol, 4-chloro-	C_6_H_5_ClO	128
22	15.508	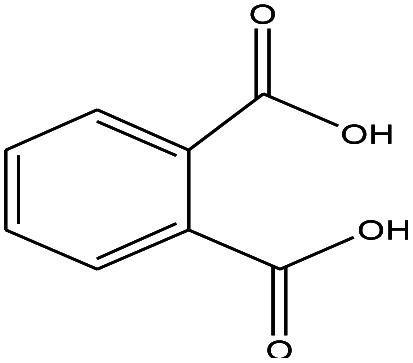	1,2-Benzenedicarboxylic acid	C_8_H_6_O_4_	166
23	20.125	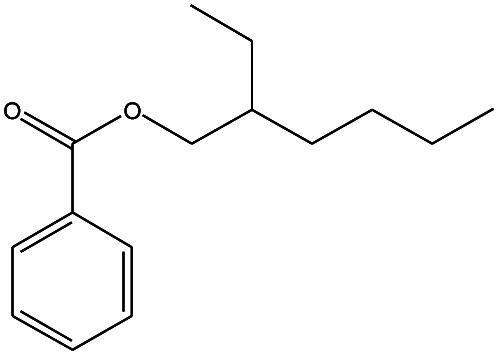	Benzoic acid, 2-ethylhexyl ester	C_15_H_22_O_2_	234
24	23.333	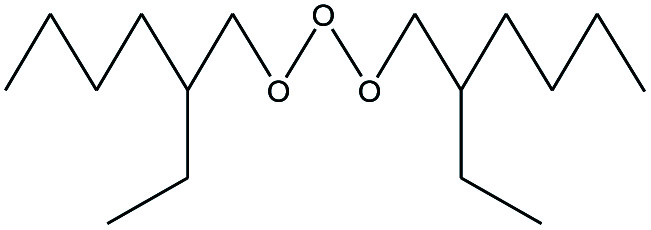	Sulfurous acid, di(2-ethylhexyl) ester	C_16_H_34_O_3_	306
25	26.433	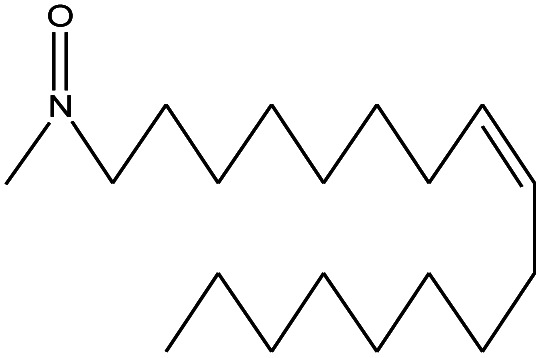	9-Octadecenamide, (*Z*)-	C_18_H_35_NO	281
26	28.177	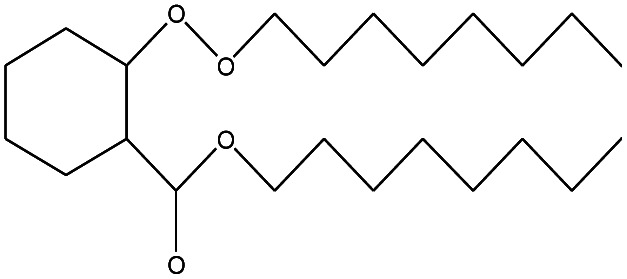	Bis(2-ethylhexyl) phthalate	C_24_H_38_O_4_	390
27	32.433	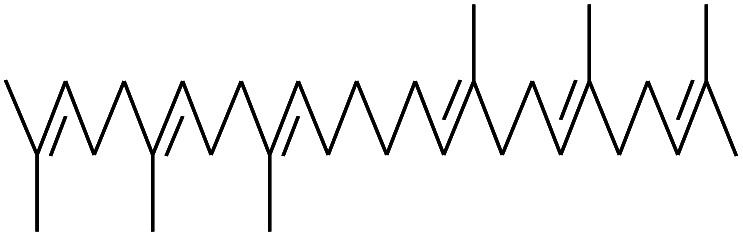	Squalene	C_30_H_50_	410
28	1.383	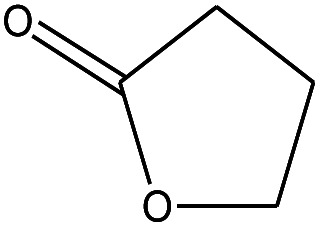	Ethane, dizao-	C_4_H_6_O_2_	86

**Table 4:** Compound identified in the Py-GC/MS of the PVC/10 wt% MH@DOPO@ MF composite

No.	Retain time (min)	Molecular structure	Name	Molecular	*M*_w_ (g mol^−1^)
1	1.383	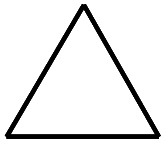	Cyclopropane	C_3_H_6_	42
2	1.500	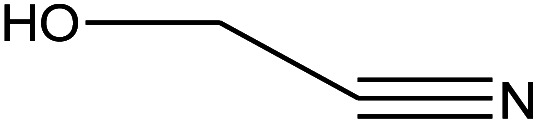	Acetonitrile, hydroxy-	C_2_H_3_NO	57
3	1.800	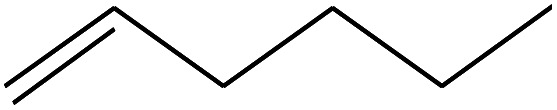	1-Hexene	C_6_H_12_	84
4	1.892	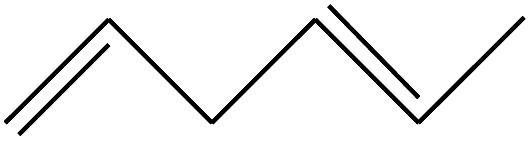	1,4-Hexadiene	C_6_H_10_	82
5	2.117	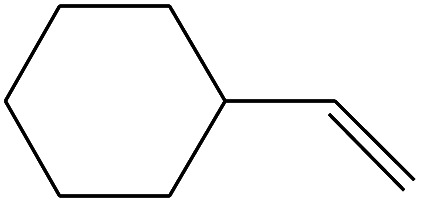	Cyclohexylacetylene	C_8_H_12_	108
6	2.258	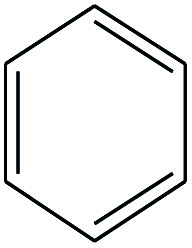	Benzene	C_6_H_6_	78
7	2.508	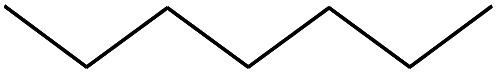	2-Heptene	C_7_H_14_	98
8	2.675	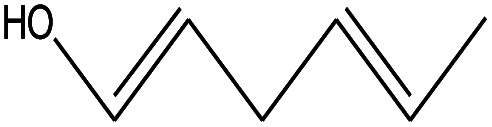	2,4-Hexadien-1-ol	C_6_H1_0_O	98
9	3.092	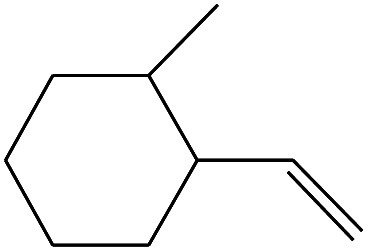	Cyclohexane, 1-ethenyl-2-methyl-, *trans*-	C_9_H_16_	124
10	3.600	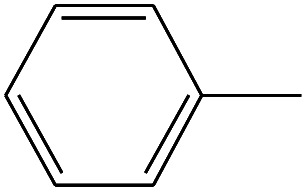	Toluene	C_7_H_8_	92
11	3.917	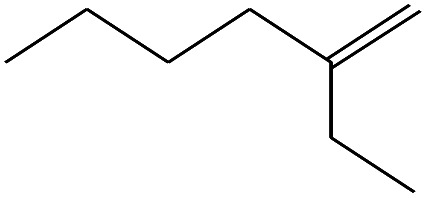	Heptane, 3-methyl-	C_8_H_16_	112
12	4.125	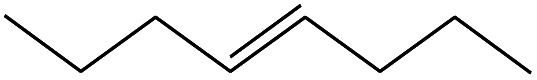	4-Octene, (*E*)-	C_8_H_16_	112
13	4.383	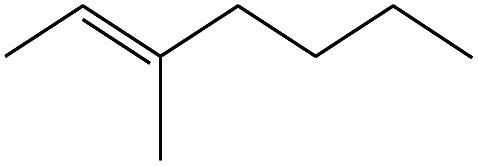	2-Heptene, 3-methyl-	C_8_H_16_	112
14	8.542	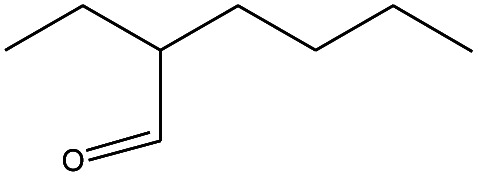	2-Ethylhexanal	C_8_H_16_O	128
15	9.875	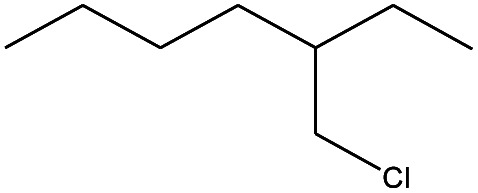	Heptane, 3-(chloromethyl)-	C_8_H_17_Cl	148
16	11.425	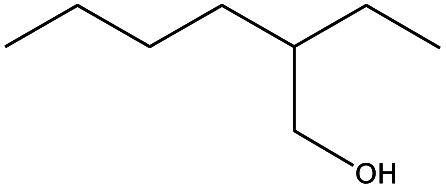	2-Ethyl-1-hexanol	C_8_H_18_O	130
17	12.925	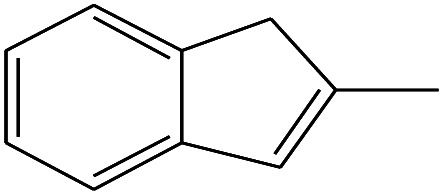	2-Methylindene	C_10_H_10_	130
18	15.692	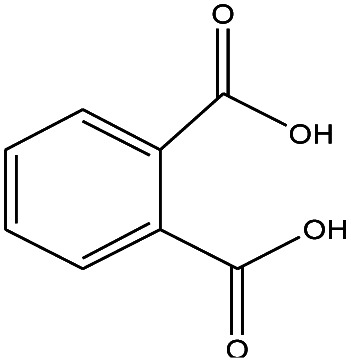	1,2-Benzenedicarboxylic acid	C_8_H_6_O_4_	166
19	20.100	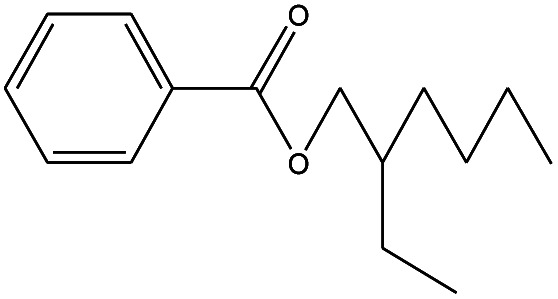	Benzoic acid, 2-ethylhexyl ester	C_15_H_22_O_2_	234
20	21.808	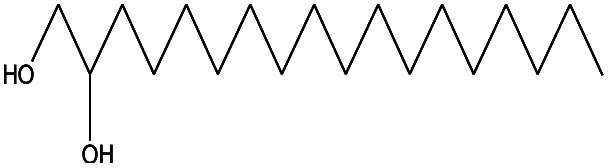	1,2-Octadecanediol	C_18_H_38_O_2_	286
21	28.142	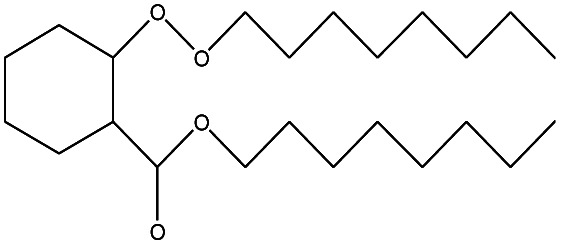	Di-*n*-octyl phthalate	C_24_H_38_O_4_	390

### 3.5. TGA-FTIR analysis

**Fig. 7:**
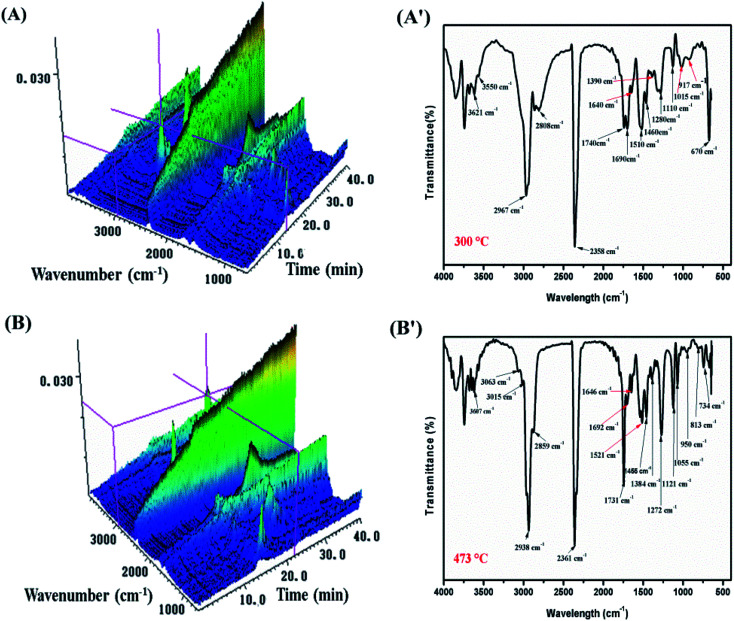
3D diagrams and TGA-FTIR spectra of the gaseous phase in the thermal degradation of pure flexible PVC, (A and A′) 300 °C min, (B and B′) 473 °C.

**Fig. 8:**
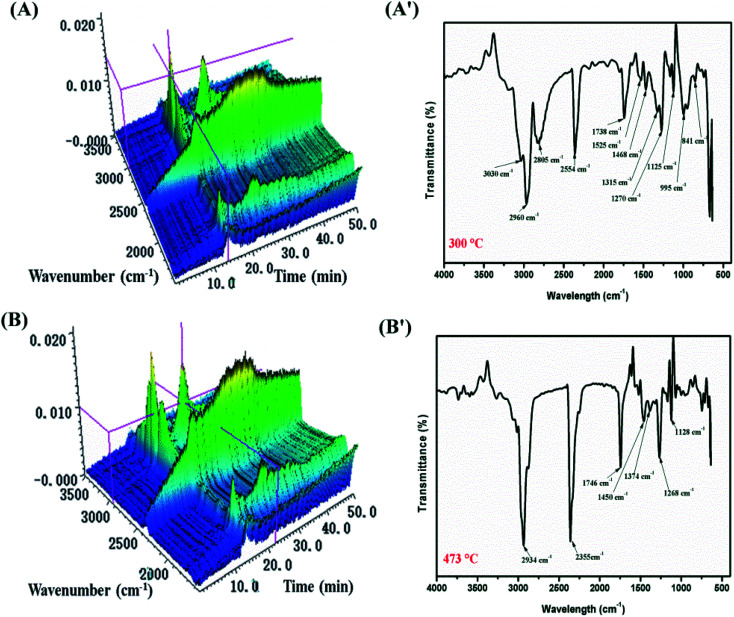
3D diagrams and TGA-FTIR spectra of the gaseous phase in the thermal degradation of PVC/10 wt% MH@DOPO@MH, (A and A′) 300 °C min, (B and B′) 473 °C.

TGA-FTIR tests were carried out in order to further understand the flame-retardant mechanism in the gas-phase of MH@DOPO@MF on the thermal degradation of PVC materials. Fig. 7 and 8 show 3D diagrams and FTIR spectra of the gaseous phase during the thermal degradation of pure flexible PVC and its PVC/10 wt% MH@DOPO@MF at different times. As shown in Fig. 7 and 8, it can be seen that the introduction of MH@DOPO@MF inhibits the generation of gaseous products during pyrolysis process. The major gas products evolved from pure flexible PVC are assigned to the characteristic peaks of water (3626 cm^−1^), aliphatic components (2967 cm^−1^, 2808 cm^−1^, 1015 cm^−1^, 1120 cm^−1^, 1273 cm^−1^), CO_2_ (2361 cm^−1^), ester or ether components (1745 cm^−1^, 1273 cm^−1^, 1120 cm^−1^, 1015 cm^−1^), aromatic compounds (1924 cm^−1^, 1514 cm^−1^, 1463 cm^−1^, 734 cm^−1^ and 813 cm^−1^), and halohydrocarbon components (2938 cm^−1^, 2808 cm^−1^, 734 cm^−1^ and 813 cm^−1^). These main characteristic peaks are well consistent with the results in the reported literature.^23,41,42^ The pyrolysis products for PVC/10 wt% MH@DOPO@MF are similar with those of pure flexible PVC, but some differences can be observed in comparison with those of pure flexible PVC. The FTIR spectra of volatile gases evolved from pure flexible PVC and its PVC/10 wt% MH@DOPO@MF at 300 °C and 473 °C are plotted in Fig. 7(A′, B′) and 8(A′, B′). It can be seen that the peak intensities of pyrolysis products of PVC/10 wt% MH@DOPO@MF are visibly higher than those of pure flexible PVC at 300 °C, for instance, aromatic compounds, ester or ether components. Nevertheless, the peak intensities of CO_2_ (2361 cm^−1^), ester or ether components (1745 cm^−1^) of PVC/10 wt% MH@DOPO@ MF are lower than those of pure flexible PVC 300 °C and 473 °C. Especially, based on the 3D images in Fig. 7(A, B) and 8(A, B), the total absorption intensity of PVC/10 wt% MH@DOPO@MF is obviously weaker than that of pure flexible PVC at 473 °C. The reduction in the total release of the organic volatiles is attributed to both of the barrier effect of phosphorus-containing char layer and MgO layer formed during the pyrolysis. From the above analysis, it is speculated that obtained MH@DOPO@MF can effectively prevent the production of vapors volatiles, because it can prevent the breaking of the carbonaceous backbone and oxidation of the unstable char. Thus, the combustion in the pyrolysis process is further restricted and the release of smoke is reduced.

### 3.6. Flame-retardant mechanism

**Scheme 2:**
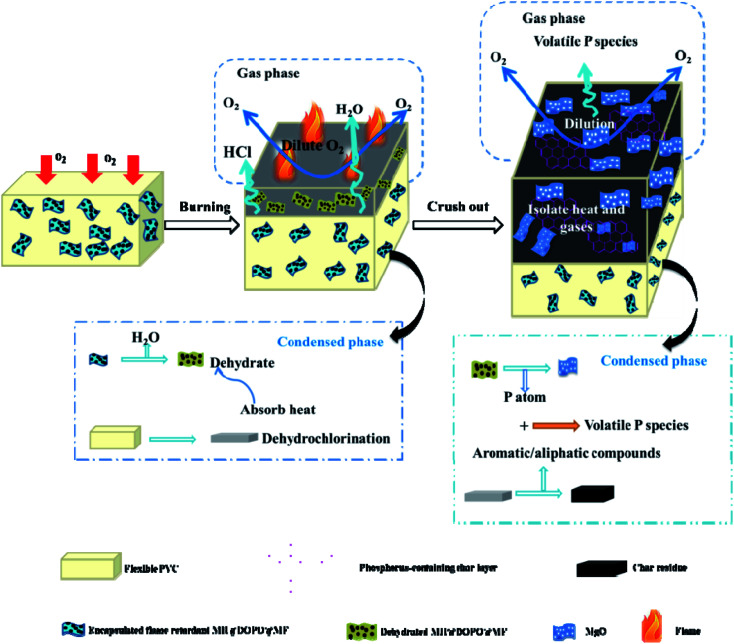
Schematic illustration of the flame-retardant mechanism of PVC/10 wt% MH@DOPO@MF.

Summarizing the above-mentioned results, the possible flame-retardant mechanism of PVC/10 wt% MH@DOPO@MH composite was proposed and schematically illustrated in Scheme 2. Pure flexible PVC decomposed and released a large amount of pyrolysis products during combustion process. Due to continuous heating and fuel supply, toxic gases (such as HCl) and heavy black smoke are released in large quantities, which is a potential fire hazard of pure flexible PVC. However, the incorporation of MH@DOPO@MF into PVC materials significantly inhibits the release of heat, smoke and toxic gases. Consequently, the high fire safety of PVC/10 wt% MH@DOPO@MF can be attributed to the followings. On the one hand, the rupture of P–C bonds in MH@DOPO@MF produces the acidic substances especially at high-temperatures such as phosphoric acid and polyphosphoric acid, that bind the vermicular carbon together, so as to reduce gaps in the carbon, which benefits to the formation of compact, continuous and intumescent char layer.^43–45^ On the other hand, the endothermic decomposition of MH in MH@DOPO@MF releases water vapor at high-temperature that can dilute the hot air and cool the surface of the pyrolysis zone, and generates MgO char residue layer with physical protection effect. Furthermore, the decomposition of MF in MH@DOPO@MF releases noncombustible gases (*e.g.* NO_*x*_) during the combustion process so as to dilute flammable gases and O_2_ concentration around the material.^11^ As a result, the core@double-shell structured flame retardant (MH@DOPO@MF) incorporated into PVC materials presents better synergistic flame retardancy and smoke suppression effects.

### 3.7. Mechanical property

Mechanical properties of pure flexible PVC, PVC/20 wt% MH and PVC/10 wt% MH@DOPO@MF composites were evaluated by tensile tests and the obtained results are listed in Table 5. The tensile strength of PVC/20 wt% MH is decreased from ∼20.87 MPa to 15.78 MPa, and its elongation at break is decreased from ∼271.25% to 168.78% in comparison with pure flexible PVC. This observed phenomenon indicates that high-loading of MH has great damage to the mechanical properties of PVC materials. Whereas, the tensile strength of PVC/10 wt% MH@DOPO@MF is decreased from ∼20.87 MPa to 19.04 MPa, and its elongation at break is decreased from ∼271.25% to 221.41% compared with pure flexible PVC. The results show that there is a very prominent synergistic flame-retardant effect among MH, DOPO and MF to PVC materials without significantly damaging their mechanical properties.

**Table 5:** Mechanical properties of pure flexible PVC, PVC/20 wt% MH and PVC/10 wt% MH@DOPO@MF

Sample	Tensile strength (MPa)	Elongation at break (%)
Flexible PVC	20.87 ± 4.53	271.25 ± 13.25
PVC/20 wt% MH	15.78 ± 3.65	168.78 ± 15.45
PVC/10 wt% MH@DOPO@MF	19.04 ± 4.87	221.41 ± 10.65

## 4. Conclusion

In summary, the as-prepared core@double-shell structured MH@DOPO@MF was incorporated into flexible PVC materials by a melt-blending method and pure flexible PVC, PVC/MH and PVC/MH@DOPO@MF composites were also prepared for comparison. The LOI value of PVC/10 wt% MH@DOPO@MF increased to 30.9% with the V-1 rating in UL-94 test. Also, its PHRR, THR, PSPR and TSP were decreased by ∼28.3%, 11.6%, 28% and 33.8% in comparison with those of pure flexible PVC. The improved flame retardance of PVC/MH@DOPO@MF is attributed to the synergistic flame-retardant effect of the MH coordination with DOPO and MF. This promotes catalytic char, resulting in a more coherent and compact char layer which inhibits the release of heat within the char. SEM images showed that MH@DOPO@MF facilitated the formation of char layer with compact and continuous structure, which can effectively insulate flammable materials and heat. TGA-FTIR results showed that the excellent smoke suppression effects of MH@DOPO@MF was associated with diluting O_2_ and flammable gases so as to prevent the chain breaking of carbonaceous backbone and the oxidation of unstable char. Based on the analysis of volatile gases and residues, the flame retardance of MH@DOPO@MF in flexible PVC materials is performed in both of the gas-phase and condensed-phase mechanisms. As for the mechanical properties of PVC/10 wt% MH@DOPO@MF, its tensile strength and elongation at break reduced 8.7%, 18.4%, respectively, in comparison with the ones of pure flexible PVC. These obtained results revealed that the core@double-shell structured MH@DOPO@MF microcapsules behaved as an environment-friendly halogen-free flame retardant and smoke suppressant.

## Conflicts of interest

There are no conflicts to declare.

## Supplementary Material

RA-012-D1RA09030E-s001
