# Supplementary material for: Synergistic effects of core@double-shell structured magnesium hydroxide microcapsules on flame retardancy and smoke suppression in flexible poly(vinyl chloride)
Source: RSC Adv. 2022 Jan 20;12(5):2914–27. doi: 10.1039/d1ra09030e (PMC8979050; doi:10.1039/d1ra09030e)
Supplement: RA-012-D1RA09030E-s001 [file RA-012-D1RA09030E-s001.pdf]

Electronic Supplementary Material (ESI) for RSC Advances.
This journal is © The Royal Society of Chemistry 2022

Electronic Supplementary Material (ESI) for RSC Advances.

This journal is © The Reyal Society of Chemistry 2022

Supporting Material for

Synergistic effects of core@double-shell structured magnesium hydroxide microcapsules on flame retardancy and smoke suppression in flexible poly(vinyl chloride)

Jingshui Xu, Haiying Yang, Zibo Luo, Dang Wu, Gengyu Cao*

In this work, we have prepared core@double-shell structured MH@DOPO@MF flame retardant and main investigate its flame retardancy and smoke suppression effects in flexible PVC. The chemical structure and morphologies of the as-prepared MH@DOPO@MF was characterized by FTIR and SEM, the results was shown in Fig.1 and Fig.2 (**Supporting Material**).

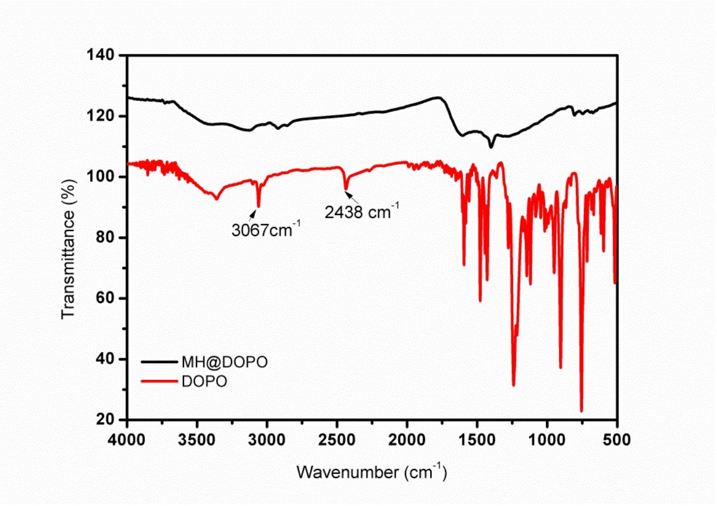

Fig.1 FTIR spectra of MH@DOPO and DOPO

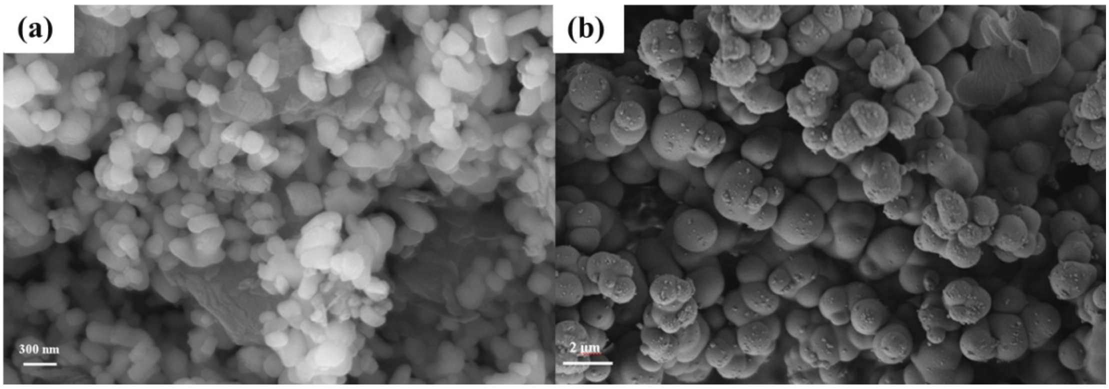

Fig.2 SEM images of (a) $Mg(OH)_2$ and (b) MH@DOPO@MF

As shown Fig.1, the FTIR spectrum of DOPO, transmittance band at 3067 cm^{-1} represents the C-H stretching vibration for benzene ring and the typical band at 2438 cm^{-1} is representive of P-H. For the FTIR spectrum of MH@DOPO, the typical band of DOPO at 2438 cm^{-1} disappeared. In Fig.2, the morphology of MH is nanoscale laminate structure, the morphology of MH@DOPO@MF is sphere and lamellar particles are not observed. Obtained results indicated that MH was encapsuled in resins.
